# Reliable Asynchronous Image Transfer Protocol in Wireless Multimedia Sensor Networks

**DOI:** 10.3390/s100301487

**Published:** 2010-02-26

**Authors:** Joa-Hyoung Lee, In-Bum Jung

**Affiliations:** Department of Computer Science and Engineering, Kangwon National University, Chuncheon Gangwondo, 200-701, Korea; E-Mail: jinnie4u@kangwon.ac.kr

**Keywords:** WMSN, image, reliability, double sliding window, cross –layer, preemption

## Abstract

In the paper, we propose a reliable asynchronous image transfer protocol, RAIT. RAIT applies a double sliding window method to node-to-node transfer, with one sliding window for the receiving queue, which is used to prevent packet loss caused by communication failure between nodes, and another sliding window for the sending queue, which prevents packet loss caused by network congestion. The routing node prevents packet loss between nodes by preemptive scheduling of multiple packets for a given image. RAIT implements a double sliding window method by means of a cross-layer design between the RAIT layer, routing layer, and queue layer. We demonstrate that RAIT guarantees a higher reliability of image transmission compared to the existing protocols.

## 1. Introduction

The recent widespread use of small, inexpensive multimedia sensors such as CMOS imaging sensors and hardware improvements to wireless sensor nodes have led to the creation of wireless multimedia sensor networks (WMSNs), which are capable of acquiring multimedia content such as sound and images. WMSNs can be used in many applications such as security programs and the monitoring of dangerous areas and can improve the quality of application services provided by networks, as compared with traditional wireless sensor networks, which transmit text-based information [1–5].

However, multimedia content has several unique characteristics as compared with text-based information, and thus requires anew transfer protocol for WMSNs. First, the size of multimedia content, especially image data, is much greater than that of text-based data. Multimedia data consist of large-sized groups of numerical values, whereas text-based data such as temperature or brightness are usually expressed as a single numerical value. For example, the temperature of a place can be expressed as 25 or 30, which requires just one or two bytes, for which a small packet is sufficient. However, one small (64 × 64) image of a place would require 4096 bytes, which in turn requires many packets to transmit because the packet size in a wireless sensor network is usually quite small, *i.e.,* from 10s∼100s bytes. Second, multimedia data is quite sensitive to data loss, whereas text-based data is relatively tolerant of data loss. The loss of a small fraction of image data leads to the discarding of the entire image or to a drastic reduction in image quality. Because multimedia content is quite large, it requires many packets that cannot be lost or dropped, if high quality is to be realized. As a consequence, in order to deal with multimedia content, a network protocol should provide end-to-end reliability of packet transmission, in consideration of the characteristics of multimedia content [6–15].

However, existing protocols in WMSN are not designed to accommodate multimedia content. These protocols are usually based on the protocols of traditional networks such as the internet, where there are frequent packet losses whenever the network becomes congested. Thus, packets with multimedia content in WMSN can be dropped in the case of congestion. Some research efforts on WMSNs have focused on congestion control, but this research does not guarantee end-to-end packet delivery. Moreover, in WMSNs, packet transmission takes place through the air, a medium in which many errors or losses can occur, so that packet transmission in WMSNs is generally regarded as quite unreliable. Some research that has claimed to provide reliable packet transmission usually seeks to reduce the packet loss or error that occurs in node-to-node transmission, not end-to-end. For this reason, a new transport protocol for WMSN should be developed to facilitate the reliable transfer of large, loss-sensitive multimedia content [16–17].

In this paper, we propose a Reliable Asynchronous Image Transfer protocol, RAIT. Our focus is upon the transfer of images, which constitute an important form of multimedia content. The proposed RAIT seeks to provide the reliable transfer of whole packets of an image from a sensor node to the sink node or gateway in a WMSN. RAIT prevents packet drop and packet loss by introducing a double sliding window. One sliding window is designed to prevent packet drop from taking place inside the node due to congestion, and the other is designed to prevent packet loss in the node-to-node transfer. For the sliding windows to work properly, the lower sensor node, which sends the image packet, must know the queue state of the upper sensor node, which receives the image packet. The sender node should stop sending packets whenever the queue of the receiver node becomes full. The network protocol should therefore be coordinated with the queuing layer. RAIT introduces a cross-layer design technique, which enables the queuing layer and network layer to work cooperatively, and based on this cooperation, a token-bucket technique is used to control the packet flow. Moreover, RAIT includes a preemption scheme that guarantees the exclusive possession of a parent node by a child node. With this preemption scheme, RAIT can not only reduce the storage space requirement but also reduce the competition among child nodes.

In the next section, we review related studies. Section 3 describes RAIT, and Section 4 provides a performance evaluation. We conclude the paper in Section 5.

## 2. Related studies

There has been little research on the design of a reliable transfer protocol for wireless sensor networks. Although there has been considerable research into reliable transport in wireless networks, such as the Ad-Hoc network, the current approaches of reliable transport protocols are regarded as being suitable for a sensor network, especially for images. PSFQ, MintRoute, and RMST are known reliable transport protocols for wireless sensor networks. These protocols are based on NACK and utilize ARQ for reliable data transport. The cost metric in MintRoute consists of a hop count to the sink node and link quality. The main purpose of the cost metric is to maximize data delivery by choosing the shortest and most reliable path to the sink node. Hop count increases reliability by reducing the number of nodes toward the sink node. Link quality also increases reliability by favoring more reliable links. These protocols can prevent packet loss caused by collision or error during node-to-node transfer, but packet drops due to congestion inside the node can still take place. Even when packets are received properly, the packet will be dropped if the sending queue is full. Error protection in image transport is well understood in conventional networks such as the Internet. Several methods have been proposed to improve the error robustness of packet transfer. Forward error correction (FEC) allows for recovery from errors by incorporating controlled redundant data [18–22].

Multipath transport has been studied in the past, in both wired and wireless networks. It has mainly been used to increase the aggregate capacity and improve the load balancing and fault-tolerance. Recently, a number of interesting proposals regarding the delivery of image and video over wireless networks using multiple paths have been introduced. The problem of allocating packets to multiple paths has been investigated in order to minimize power consumption and end-to-end image distortion. However, it requires continuous monitoring of path quality at each hop and the reporting of this information to the source node, which makes it difficult to utilize in sensor networks. It is necessary to discuss why multipath transport schemes are not suitable for wireless sensor networks. First, as presented in previous studies, multipath transport is mainly used to combat wireless link errors through path diversity. Network congestion, which may be common in sensor networks, is not considered. Second, multipath transport schemes, which incorporate end-to-end error control, typically split data at the source and combine data from different paths at the destination. However, the intermediate nodes are unaware of the errors inside packets. Thus, the errors accumulate as the packet travels toward its destination (*i.e.,* error propagation). Above all, sending the same packets from an image through multiple paths increases energy consumption in proportion to the number of paths; a very limited critical resource in wireless sensor networks [23–27].

Obviously, the use of FEC coding alone cannot also address the problem of network congestion in which the corrected packet is dropped. Furthermore, FEC requires redundant data to be transferred as an error check and correction, which increases the number of packets required, given the large number of packets that images require [28,29].

## 3. RAIT

We consider a densely deployed wireless sensor network that includes camera-equipped nodes. A camera-equipped node frequently transfers an image to a sink node or gateway. A tree-based routing protocol is used to construct the path from the sensor nodes to the sink node. The path to the sink node can be changed whenever the link states to the upper nodes are unreliable due to node failure or obstacles. The lower nodes in a routing tree have to compete with other nodes for an upper node in a routing tree. The errors and loss that take place in the air are corrected by an error-correcting code such as FEC or ACK, which are based on a retransmission technique. Each sensor node manages two queues: one for receiving and the other for sending [28,29].

**Figure 1.**
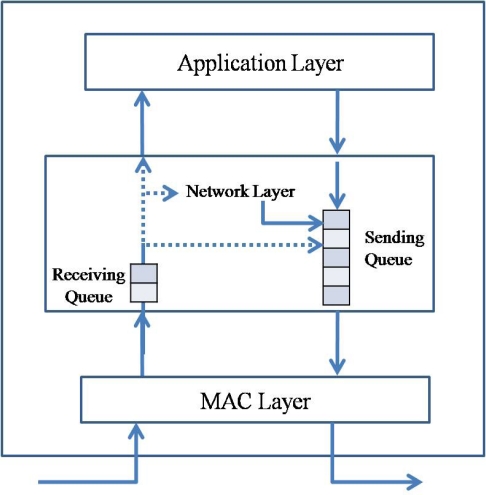
Packet flow in a sensor node. A sensor node has two queues, receiving queue for received packets and sending queue for generated packets and transit packets.

Figure 1 presents the queuing model in a sensor node. A sensor node has two queues, a receiving queue for transit traffic and a sending queue for transferring data. The packets generated by the sensor node itself are inserted directly into the sending queue. A sensor node could generate a data packet for sensed data in an application layer or a routing packet for routing information in a network layer. Therefore, the packets received from the network also can be one of two packet types, a data packet or a routing packet. A packet received from the network is inserted into the receiving queue and then classified by the packet type and the destination address in the packet header. If the destination address of a data packet is the receiving node, the packet is transferred to an upper application layer. If the destination address is not the receiving node, the packet is inserted into the sending queue. A routing packet is used by the routing protocol in a network layer.

In the conventional network, a node works as a packet generator (consumer) or a router, however, in the sensor network, a sensor node takes charge of both the packet generator and the router, because the sensor node has to relay the packet from other sensor nodes to the sink node. In the sensor network, the sending queue in the sensor node is shared by the node itself and the child nodes of it, whereas the sending queue in the conventional network is only used by the node itself. Therefore, the network layer in a sensor node should take into account both the state of the receiving queue and the sending queue, whereas the network layer in the conventional network should take into account only one of the sending queue or the receiving queue. As a consequence, one sliding window algorithm is enough for the conventional network, however one more sliding window for the sending queue is required in the sensor network. In this paper, we propose the double sliding window algorithm for the reliable transfer in the sensor network.

### 3.1. Sliding Window

**Figure 2.**
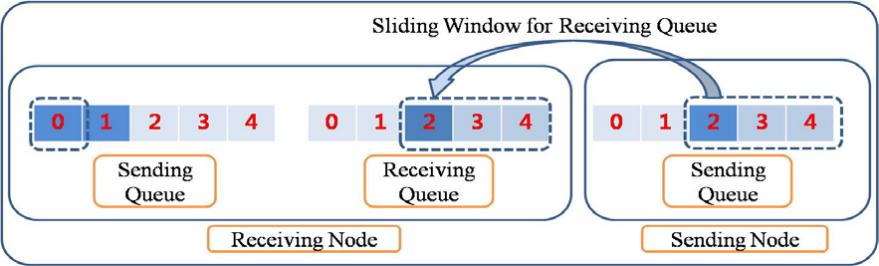
Sliding window for receiving queue. Sliding window for receiving queue limits the number of packets that the sender can send at once.

Sliding window algorithm is a method used for flow control in network transfer. A sliding window algorithm places a queue (buffer) between nodes. The sender creates a sequence number for each packet as it is transferred. Throughout the communication, it maintains the send window size so that it does not overflow. When a packet arrives in the receiver, the sliding window size for the receiving queue is decreased. When the packets in the receiving queue are retrieved by the upper network layer, the sliding window size is increased. The retrieved packets, which are delivered to the upper applications if the receiving node is the sink node, are put into the sending queue of the receiving node - provided there are available spaces in the sending queue, or they are simply dropped if the sending queue is full of packets. The sliding window for the receiving queue in RAIT functions in the same way as those in traditional networks like the Internet. Figure 2 shows an example of a sliding window for a receiving queue with a size of 5. In general, a receiving queue with a size of 1 is used in a wireless sensor network because the sensor node normally has very limited memory size.

As mentioned earlier, even if a packet is properly received in the receiving queue, the upper network layer can drop the packet in the case that the sending queue is full of previous packets. This situation can take place for many reasons, such as network congestion or link failure to an upper node. In such cases, general congestion control protocols are applied, which usually drop the overflow packets and employ a back-pressure technique to reduce the sending rate. As the back pressure propagates through the lower nodes in the routing tree the sending rate is reduced, and as a result, the congestion is eased gradually. However, it is very hard to evenly distribute the available bandwidth of a node to the lower nodes, so there can be imbalanced packet transmission among the lower nodes. Moreover, dropped packets in congested nodes have to be retransmitted by the source nodes to guarantee end-to-end reliability, which requires the consumption of additional energy.

**Figure 3.**
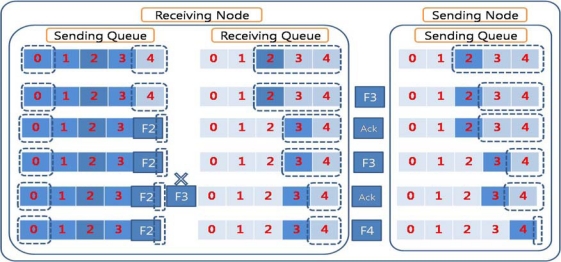
Packet drop inside node. Although a packet is properly received, the packet can be dropped if the sending queue is full.

Figure 3 illustrates packet dropping due to queue overflow in the sending nodes. The receiving queue in the receiving node has enough space for the sliding window to send an ACK packet for the received packet. The sender node therefore continues to send successive packets. Because there is no space in the sending queue in the receiving node, the retrieved packets from the receiving queue are dropped. Thus, while the dropped packets need to be retransmitted, this retransmission is not easily done because ACKed packets are emitted from the sending queue. If the dropped packets were generated from the sender node, it might retransmit the packets if they were stored elsewhere, such as in an upper layer buffer, which would require additional storage space. In the case that the sender node and source node are different and the sender node is just relaying the packets, the sender cannot retransmit the dropped packets because it has emitted the packets. The source node should be notified of the fact that some packets have been dropped. Some congestion control protocols or reliable transfer protocols use this scheme. However, the sink node has to inform the source node about the packets dropped and must wait until the packets retransmitted by the source arrive at the sink node. This requires storage space in the sink node to save the received packets, as well as a time delay.

**Figure 4.**
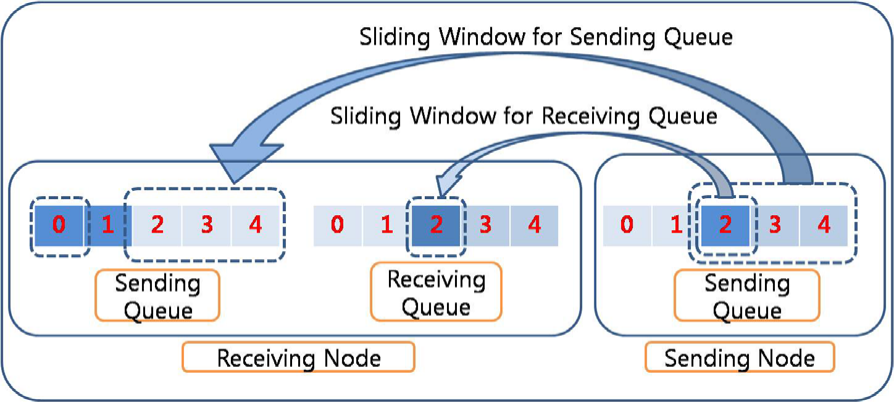
Sliding window for sending queue. Sliding window for sending queue prevents the packet drops due to the overflow of sending queue.

In RAIT, to prevent this effect, an additional sliding window is used for the sending queue in the receiving node, as shown in Figure 4. The sliding window for the sending queue is based on the available free space in the sending queue in the receiving node. The operation of the sliding window for the receiving queue is limited by the sliding window for the sending queue. The sender node cannot send a packet even if there are free spaces in the receiving queue in the case that the sliding window for the sending queue has reached its limit. This could prevent packet drop, as shown in Figure 3. The sliding window for the receiving queue still works with ACK packets. An ACK packet might be used for the sliding window for the sending queue, but this could cause several problems. If an ACK packet is sent after the successful insertion of a packet into the sending queue, the receiver might have to wait for an unpredictable period of time due to the processing time in the network layer of the receiving node. An ACK packet in the receiving queue is sent quite quickly so that the sender can also transmit the packet quickly. However, if an ACK packet is sent in the sending queue, more time is required, which implies that the sending rate is decreased.

### 3.2. Crossing Layer

**Figure 5.**
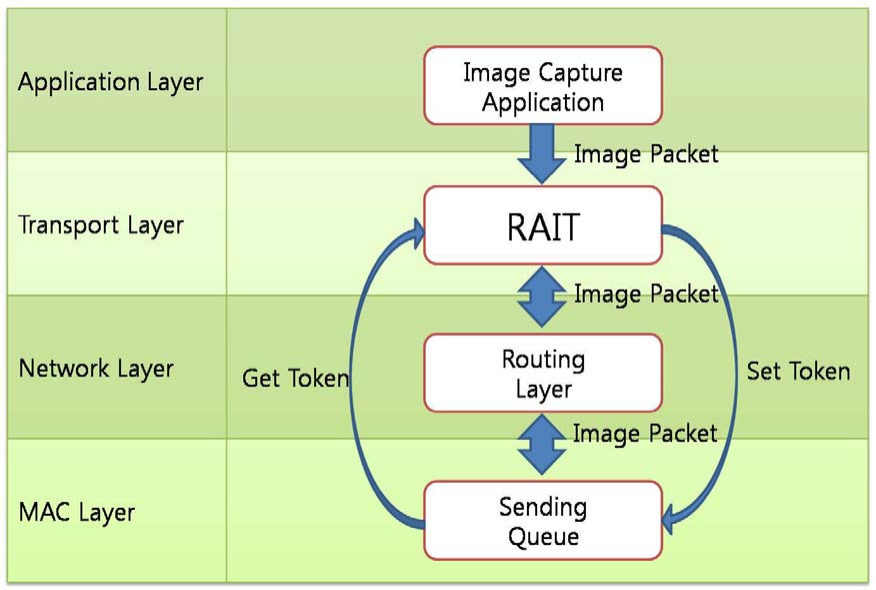
Cross-layer design. RAIT controls the sending queue with Set Token and Get Token.

In RAIT, a token-bucket mechanism is used for the sliding window for the sending queue. The sending node requests tokens from the receiving node whenever there are packets to send but it has no token. The receiving node checks the queue state and replies to the sending node with the available queue size. The sending node controls the queue state on its own, limiting the number of packets that it can send within its token limits. However, each layer in a communication protocol layer usually operates independently and is unaware of the state of other layers, and is not allowed to control other layers directly. For this reason, the transport protocol in which RAIT works should not control the operation of the queuing layer. RAIT addresses this problem by applying a cross-layer design scheme, which is used in sensor networks on occasion. The protocol layers working in a sensor node can be tightly coupled with each other because the sensor node has very limited resources. This scheme is called Cross-Layer Design [23]. Figure 5 shows the cross-layer design in RAIT. RAIT controls the sending queue to get the available token or set the token limit.

**Figure 6.**
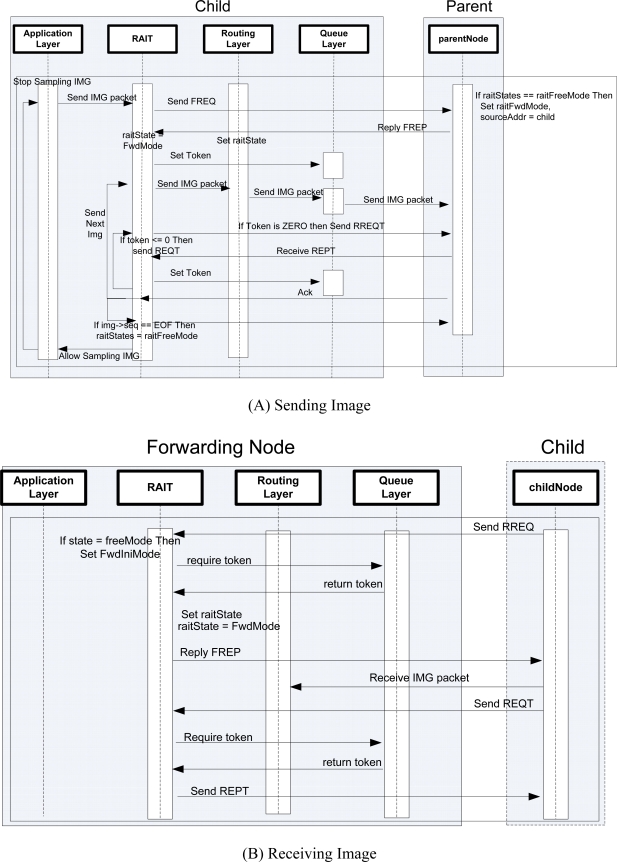
Sequence Diagram of RAIT.

Figure 6 presents the sequence diagram of RAIT:

1. Application layer takes an image and requests that the RAIT layer sends an image.
2. RAIT layer sends token request to parent node (receiving node) in routing tree.
3. RAIT layer in parent node (receiving node) checks the queue state.
4. Queue layer in receiving node returns the available queue size as a token.
5. RAIT layer in receiving node replies with a token to RAIT layer in sending node.
6. If the replied token has a value of 0 (zero), then the sending node waits for a random time period and tries again.
7. If replied token has a value larger than 0 (zero), then the RAIT layer sends a new token to queue layer.
8. Whenever queue layer sends a packet, it decreases token by 1.
9. If available tokens becomes 0 (zero), then queue layer notifies the RAIT layer.
10. Notified RAIT layer sends new token request to receiving node.
11. Until RAIT receives new token from receiving node, queue layer cannot send a packet.

### 3.3. Preemption Scheme

**Figure 7.**
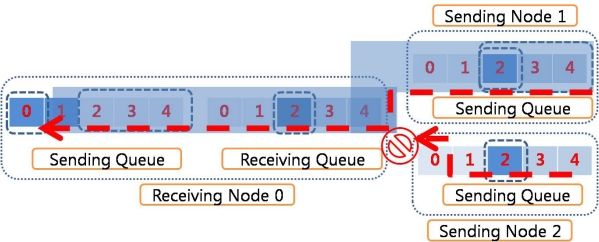
Preemption scheme. A sensor node possesses exclusively the parent node during the image transmission.

For the sliding window of the sending queue to work properly, the sending node should not change the parent node (upper node) until it consumes all the tokens received from the upper node, because the receiving node would not allow another child node (lower node) to send a packet until it receives as many packets as it has granted tokens. This means that RAIT guarantees that a sending node will transfer as many packets as it has granted tokens. In addition, RAIT also guarantees that a node will preempt an upper node, as shown in Figure 7. Here, preemption means exclusive possession. If a node is sending packets of an image to an upper node, other nodes that share the upper node with the sending node (child nodes of the receiving node), should wait for the sending node to complete sending all the packets of an image. For example, in Figure 7, node 0 is a parent of node 1 and node 2. If node 1 is sending packets to node 0, node 2 has to wait for node 1 to complete all its packets. If the size of the sending queue in the sensor node is smaller than the image size, then an image would occupy several nodes at once. For example, if a sensor node has a queue that is size 10 and an image that consists of 30 packets, then the image would occupy 3∼4 sensor nodes.

**Figure 8.**
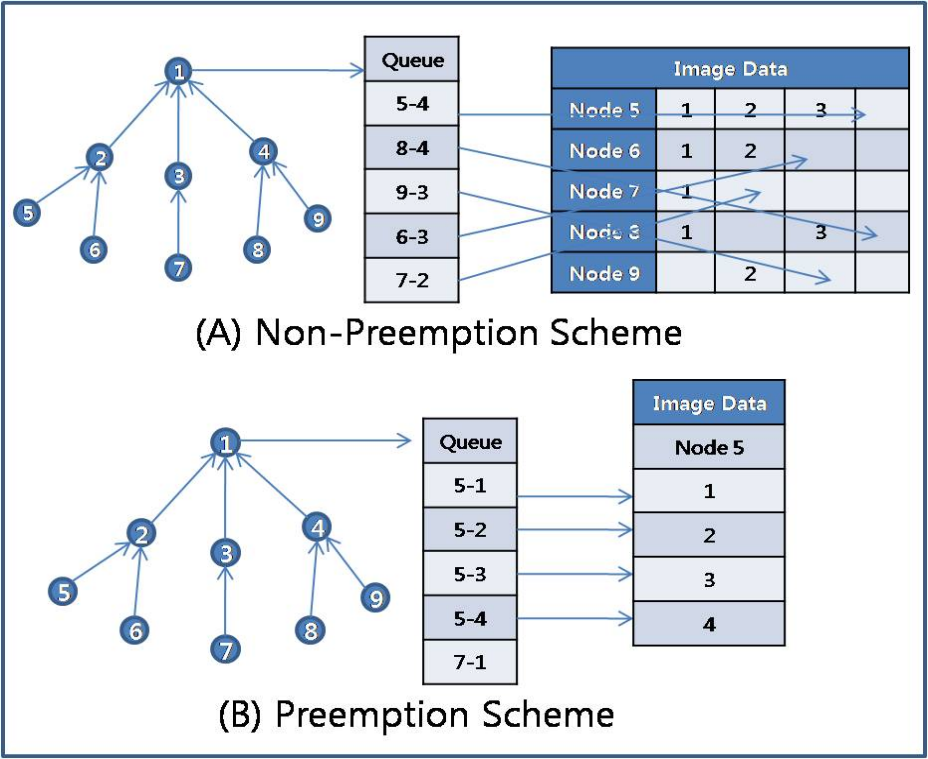
Management of received image packets in sink node. With non-preemption scheme (A), sink node has to manage the memory space in proportion to the number of nodes, whereas with preemption scheme (B), sink node has to manage the memory space with size of image.

The preemption scheme in RAIT can reduce the necessary memory spaces in the sink node that collects and arranges image packets received from the sensor nodes. If a non-preemption scheme is used in which sensor nodes compete to send a packet to a parent node, the sink node has to allocate memory space according to the number of sensor nodes, as shown in Figure 8 (A), because packets from several sensor nodes have arrived at the same time. Moreover, packets from a sensor node could arrive at a sink node in a sequence different from their original sequence, because packets would be transferred through multiple paths if the sensor nodes frequently change their parent node in a non-preemption scheme. In this case, a packet could arrive at a sink node earlier than other packets with a higher sequence number, so that a sink node would have to distinguish between a delayed packet and lost packet. For example, in Figure 8 (A), a sink node receives packets 1 and 3, skipping packet 2 from node 8. In this case, the sink node has to distinguish whether packet 2 has been lost somewhere or has just been delayed. The sink node has to wait for packet 2 for an uncertain period of time, and if it does not arrive in that time, then the sink node can regard packet 2 as lost. The problem is that it is hard to define the waiting time for a delayed packet. Even more serious is the problem of how to treat a lost packet. If the sink node disregards the lost packet, then the quality of the image would be degraded to the extent of the information in the lost packets. If the sink node requests that the source node of the lost packet retransmit the lost packets, then the sink node must wait for the retransmitted packet to arrive, while storing the received packets somewhere in memory. This would require additional memory spaces for the delayed collection of the image [26].

On the other hand, if a preemption scheme is used, all the packets of a image arrive at the sink node together, so that a sink node can store memory for an image using much less than is needed for the non-preemption scheme, as shown in Figure 8 (B). With the preemption scheme, the storage space consumed for images in the sink node can be uniform, whereas with the non-preemption scheme, the storage space consumed would increase in proportion to the sensor nodes. Given the arrival of packets in correct sequence and no delayed packets, the preemption scheme does not require the additional memory space for delayed packets that would be required in the non-preemption scheme.

**Figure 9.**
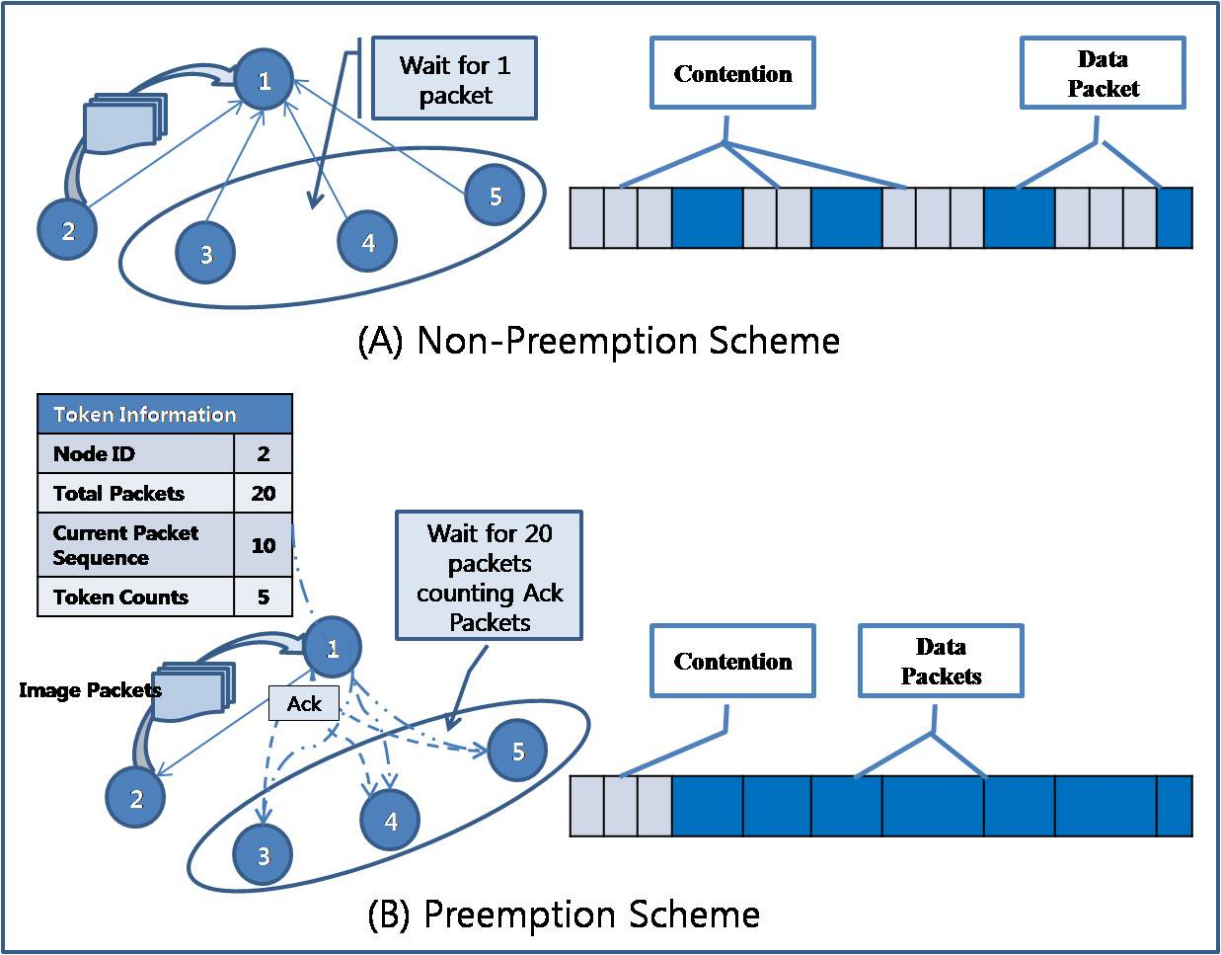
Competition for communication medium. With non-preemption scheme (A), sensor nodes have to compete for the channel every time for packet transmission, whereas with preemption scheme (B), sensor nodes just compete once.

Moreover, the competition for a communication channel among sensor nodes that try to send a packet to an upper node could be reduced by preemption in RAIT. We assume a wireless sensor network with a CSMA-based MAC protocol in which each sensor node has to compete with the others for a communication channel. Given a CSMA-based MAC protocol with a non-preemption scheme, a sensor node that attempts to send a packet will check the communication channel by sensing the carrier signal to see whether the other sensor node is already sending a packet. If there is no carrier signal, then the sensor node can send a packet. However, if a carrier signal is sensed, this means that the other sensor node has occupied the channel so that the sensor node has to wait for some time. In this case, the sensor node must check the signal after a waiting period equal to the transmission time of one packet. If there are several sensor nodes that have a packet to send, then these sensor nodes have to compete for the channel for some time. For example, as shown in Figure 9 (A), if sensor node 2 is sending a packet, then the other sensor nodes (sensor nodes 3, 4, 5) have to wait for sensor node 2 to finish sending a packet.

On the other hand, with a preemption scheme, if a sensor node is sending packets of an image, other sensor nodes that have the same parent node can know how many packets the sending sensor node is sending due to token information sent by the parent node. A token packet from a parent sensor node consists of the following information: Node ID (sending node), Total Packets, Current Packet Sequence, and Token Counts. All the child sensor nodes of the parent sensor node can receive the token packet that is broadcast by the parent sensor node, and thus the other sensor nodes can know who is sending, how many packets are being sent, and how many packets remain. These other sensor nodes will therefore wait for all the packets of an image to be transferred, not a single packet, because RAIT guarantees exclusive possession of the parent node. This means that other sensor nodes do not need to check the channel for a carrier signal, which in turn means reduced competition for a channel. Instead, these sensor nodes will listen for an ACK packet from the parent node, which sends an ACK packet whenever it properly receives a packet from a child sensor node. By counting the received ACK packets, other sensor nodes can know how many packets have been sent and how long they will need to wait. For example, as shown in Figure 9 (B), if sensor node 2 is sending 20 packets to parent sensor node 1, the other sensor nodes (sensor nodes 3, 4, 5) will wait for sensor node 2 to finish transferring 20 packets by counting until 20 ACK packets have been received. As a consequence, sensor nodes with a non-preemption scheme compete for the communication channel for a packet, whereas sensor nodes with a preemption scheme compete for the communication channel for an image.

### 3.4. Theoretical Analysis

For the data transmissions from a child sensor node to a parent node, CSMA is used to reserve the channel. We assume that each node knows the number of contending neighbor nodes (m) and contends the channel with the optimal probability p = 1/m. The probability that one contending node wins the channel is p_succ_ = (1 − 1/m)^m−1^. Since the number of slots needed until the successful reservation is a geometric random variable, the average number of contending slots (ACS) is given by

(1) $$\mathrm{ACS}=\frac{1}{{\left(1-\frac{1}{M}\right)}^{M-1}}$$

where M is the Average number of Neighbor nodes.

Assuming that the time length of the contention slot is T_s_, we have the energy consumption (CE) for channel contention:

(2) $$\mathrm{CE}=T_{s}\times P_{t}\times ACS=T_{s}\times P_{t}\times \frac{1}{{\left(1-\frac{1}{M}\right)}^{M-1}}$$

where P_t_ is the transmission power.

Assuming that the average number of packets of an image is NPI, the average total ACS to send an image one hop (IACS_one_) without RAIT is given by

(3) $$\mathrm{IAC}S_{\mathrm{one}}=NPI\times ACS=NPI\times \frac{1}{{\left(1-\frac{1}{M}\right)}^{M-1}}$$

and the average CE to send an image one hop (ICE_one_) without RAIT is given by

(4) $${\mathrm{ICE}}_{\mathrm{one}}=NPI\times CE=NPI\times T_{s}\times P_{t}\times \frac{1}{{\left(1-\frac{1}{M}\right)}^{M-1}}$$

The average total ACS to send an image to the sink (IACS_sink_) without RAIT is given by

(5) $$\mathrm{IAC}S_{\text{sin}\;k}=\sum_{i=1}^{H}(NPI\times ACS)=\sum_{i=1}^{H}\left(\mathrm{NPI}\times \frac{1}{{\left(1-\frac{1}{M}\right)}^{M-1}}\right)$$

and the average CE to send an image to the sink (ICE_sink_) without RAIT is given by

(6) $$\mathrm{IC}E_{\text{sin}\;k}=\sum_{i=1}^{H}(NPI\times CE)=\sum_{i=1}^{H}\left(\mathrm{NPI}\times T_{s}\times P_{t}\times \frac{1}{{\left(1-\frac{1}{M}\right)}^{M-1}}\right)$$

where H is the hop count from the source node to the sink node.

Assuming that the number of sensor nodes in the sensor network is N, the average total ACS to send whole images to the sink from N sensor nodes (IACS_total_) without RAIT is given by

(7) $${\mathrm{IACS}}_{\mathrm{total}}=\sum_{i=1}^{N}{\mathrm{IACS}}_{\text{sin}\;k}=\sum_{i=1}^{N}\sum_{i=1}^{H}\left(\mathrm{NPI}\times \frac{1}{{\left(1-\frac{1}{M}\right)}^{M-1}}\right)$$

and the average CE to send whole images from N sensor nodes to the sink (ICE_total_) without RAIT is given by

(8) $${\mathrm{ICE}}_{\mathrm{total}}=\sum_{i=1}^{N}{\mathrm{ICE}}_{\text{sin}\;k}=\sum_{i=1}^{N}\sum_{i=1}^{H}\left(\mathrm{NPI}\times T_{s}\times P_{t}\times \frac{1}{{\left(1-\frac{1}{M}\right)}^{M-1}}\right)$$

On the other hand, IACS_one_ with RAIT is same with the ACS and ICE_one_ is also same with CE because a sensor node with RAIT has to contend just one time and could send whole packets of an image to the parent node without interference.

The IACS^RAIT^_sink_ with RAIT is given by

(9) $${\mathrm{IACS}}_{\text{sin}\;k}^{\mathrm{RAI}T}=\sum_{i=1}^{H}\mathrm{ACS}=\sum_{i=1}^{H}\frac{1}{{\left(1-\frac{1}{M}\right)}^{M-1}}$$

and ICE^RAIT^_sink_ with RAIT is given by

(10) $${\mathrm{ICE}}_{\text{sin}\;k}^{\mathrm{RAI}T}=\sum_{i=1}^{H}\mathrm{CE}=\sum_{i=1}^{H}\left(T_{s}\times P_{t}\times \frac{1}{{\left(1-\frac{1}{M}\right)}^{M-1}}\right)$$

IACS^RAIT^_total_ with RAIT is given by

(11) $${\mathrm{IACS}}_{\mathrm{total}}^{\mathrm{RAI}T}=\sum_{i=1}^{N}{\mathrm{IACS}}_{\text{sin}\;k}^{\mathrm{RAI}T}=\sum_{i=1}^{N}\sum_{i=1}^{H}\left(\frac{1}{{\left(1-\frac{1}{M}\right)}^{M-1}}\right)$$

and ICE^RAIT^_total_ with RAIT is given by

(12) $${\mathrm{ICE}}_{\mathrm{total}}^{\mathrm{RAI}t}=\sum_{i=1}^{N}{\mathrm{ICE}}_{\text{sin}\;k}^{\mathrm{RAI}T}=\sum_{i=1}^{N}\sum_{i=1}^{H}\left(\mathrm{NPI}\times T_{s}\times P_{t}\times \frac{1}{{\left(1-\frac{1}{M}\right)}^{M-1}}\right)$$

## 4. Performance

### 4.1. Evaluation Environment

To evaluate the performance of the proposed RAIT, we simulated MintRoute [13], SRI [23] and RAIT together in TOSSIM, which is a simulator for TinyOS. TinyOS is an operating system developed for event-based sensor networks at UC Berkeley. TOSSIM provides a simulation environment that simulates a real sensor network with TinyOS. Because an application running on TOSSIM can be run on real sensor nodes such as a micaz or a telos, it can be said that our implemented simulation truly reflects the real world. In the simulation, 144 sensor nodes with 20 image sensor nodes were set to send a total of 10 frames of an image with a size of 54 packets. Each sensor node had a receiving queue with a size of 1 packet and a sending queue with a size of 32 packets [30].

Within TinyOS, MintRoute is the standard routing protocol. MintRoute is a proactive routing protocol in which sensor nodes send routing messages periodically to notify their local states. MintRoute does the best effort in transmitting packets by selecting a parent node with highest link quality. However, MintRoute does not guarantee the transmission in case of congestion. SRI is an image transmission protocol based on the wavelet transform. SRI assigns the priority to the packets based on the wavelet stage and drops packets with lower priority packets first in case of the congestion. With SRI, an image can be reconstructed with lower resolution even if some packets of the image are lost in the network.

We selected three performance evaluation matrices: total reception ratio of image, total reception number of packets, and average transmission duration of an image. “Total reception ratio of image” means how many images out of 10 images sent by sensor nodes arrive at a sink node. If one or more packets were lost out of 54 packets for an image, that image was regarded as lost. “Total reception amount of packets” means the total number of packets received by the sink node. “Average transmission duration of an image” means the average time it takes for all the packets of an image to be transmitted from the source sensor node to the sink node.

We varied the number of image-sending nodes from 1 to 20 and the time synchronization range from 0 to 40 s. If there is only one sending sensor node then there is no competition or congestion, and all the packets of an image will arrive at a sink node. On the other hand, if two or more sensor nodes try to send images, there will be competition and congestion, and as a consequence, some sent packets will be lost. The more sensor nodes there are trying to send images, the more packets will be lost. In addition, we also changed the time synchronization range among the sensor nodes. Time synchronization refers to the relative difference in the clocks among sensor nodes in a sensor network. If the clocks of the sensor nodes are synchronized very precisely, every sensor node tries to send an image simultaneously. In the simulation, 0 s synchronization meant that the sensor nodes were perfectly synchronized. As the synchronization range was increased, the sensor nodes’ transmission of an image became more random. We varied the time synchronization range to see the effects of competition and congestion. The more sensor nodes there were trying to simultaneously send images, the more competition and congestion took place and thus the more packets that were lost during transmission.

### 4.2. Variation of Number of Sending Nodes

#### 4.2.1. Reception ratio

The probability of network traffic congestion can increase in proportion to the number of sending nodes in a network. Each added increment of network congestion would increase the probability of packet loss, which would lead to the loss of images. As a result, the number of sending nodes can have an influence on the received images. In this simulation, the number of image-sending nodes was set at 1, 2, 4, 8 and 20. The sending queue size was fixed at 32 and the sensor nodes were synchronized within 40 s. With this setup, we evaluated the total packet reception ratio and total image packet reception ratio.

**Figure 10.**
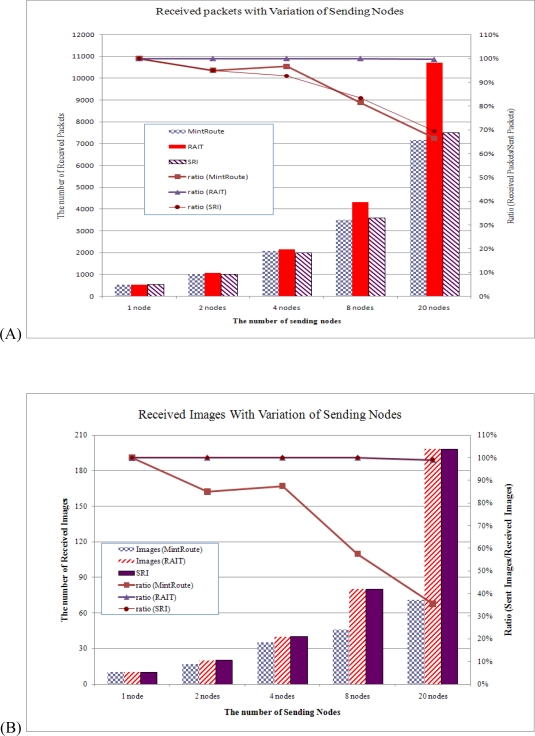
Number of received packets (A) and number of received images (B) with variations in number of sending nodes. RAIT collected whole packets of transferred images while the reception ratio with MintRoute and SRI was decreased in an inverse proportion to the number of sending nodes.

Figure 10 shows the number and ratio of received packets and images with variations in the number of sending nodes. In Figure 10 (A), bar graphs show the number of received packets with variations in the number of image-sending nodes, and the line graphs show the ratio computed by dividing the number of total received packets by the number of total sent packets. When only one sensor node sent images, MintRoute showed a 100% ratio of packet reception, but the packet reception ratio decreased even with two sensor nodes in MintRoute and SRI. As the number of sending nodes was increased, the packet reception ratio in MintRoute and SRI decreased drastically. When 20 sensor nodes were sending images, about 3,000 packets were lost, which was 33% of the sent packets. This result demonstrates that with MintRoute and SRI, many packets were lost due to network congestion caused by images. On the other hand, with RAIT, all sent packets were received by a sink node, showing a 100% reception ratio, even though the number of sending nodes was increased.

Figure 10 (B) shows this difference in performance even more distinctly. When we classified images with one or more lost packets, the image reception ratio in MintRoute decreased more drastically. Although more than 90% of the sent packets were received by a sink node, the image reception ratios were about 85% with MintRoute. Given 20 sending nodes, only 70 out of 200 images were received (35%), even though about 67% of the packets were successfully received. 30% of the packets were lost during transmission and even worse, 30% of the packets were dropped at the sink node. Lost or dropped packets are directly related to the wastage of energy in sensor nodes because packet transmission consumes a great amount of energy, which is a very limited resource. With MintRoute, the reliability of image transmission was not only guaranteed, the energy efficiency was also decreased. On the other hand, RAIT and SRI showed a 100% image reception ratio regardless of the number of sending nodes. With SRI, the image reception ratio was 100% even if the packet reception ratio was decreased. An image that was transformed with wavelet can be reconstructed by wavelet transform with lower resolution even if some packets were lost. Therefore, the image reception ratio is high even if the packet reception ratio is low. With RAIT, the image source nodes or relaying nodes could exclusively possess the parent nodes during transmission of all the packets of an image, so that there could be no network congestion, ensuring that all sent images were received.

#### 4.2.2. Transmission duration

**Figure 11.**
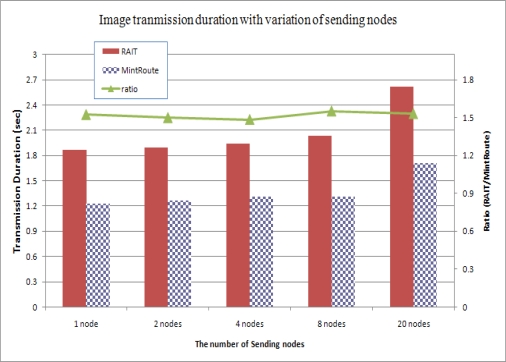
Image transmission duration with variations in number of sending nodes

While RAIT can guarantee the reliability of image transmission with a preemption scheme and double sliding windows, these schemes increase the duration of image transmission. To see the effect in terms of duration, we estimated the average transmission duration of images. Figure 11 shows the average transmission duration of images with variations in the number of sending nodes.

As shown in Figure 11, the image transmission duration with RAIT took 0.6 s more than MintRoute, even with one sending node. This could be analyzed as overhead of RAIT. However, this overhead does not increase with an increase in the number of sending nodes. In the case of 20 sending nodes, the duration increased up to 2.6 s with RAIT and 1.7 s with MintRoute. This amounted to about a 40% increase in both RAIT and MintRoute, showing a ratio similar to that shown in Figure 11, while the total number of transferred packets with RAIT was 33% larger than that of MintRoute, showing a large difference in the ratios shown in Figure 9. As a consequence, with RAIT, the transmission duration of an image can increase, but this would not be greatly affected by an increase in sending nodes. We might attribute this result to the effect of the preemption scheme explained in Section 3.3. With a preemption scheme in RAIT, the competition among child sensor nodes with the same parent sensor node can be greatly reduced because the preemption scheme guarantees the exclusive possession of a parent node. Given such reduced competition, the number of collisions between packets can also be decreased, and the sensor node that exclusively possesses the parent node can send successive packets without interruption or disturbance from other nodes. As a result, each packet can be transferred more quickly in RAIT than in MintRoute and SRI.

#### 4.2.3. PSNR Comparison

**Figure 12.**
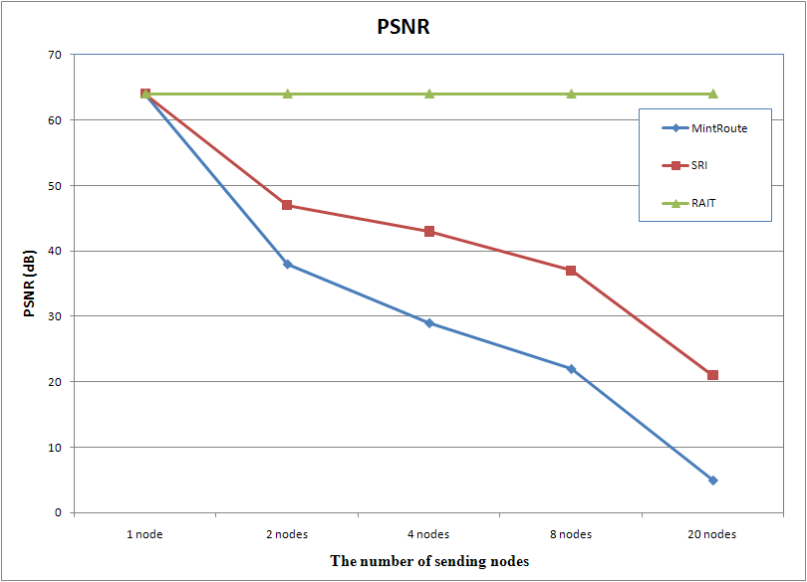
PNSR. RAIT presents uniform PSNR while PSNR of MintRoute and SRI was decreased very much.

PSNR (Peak Signal-to-Noise Ratio) presents the ratio between the maximum power of a signal and the power of corrupting noise. The PSNR is most commonly used as a measure of quality of reconstruction of lossy codecs such as image compression. High PSNR means high image quality, and thus image transmission protocols should guarantee high PSNR. Figure 12 presents the PSNR comparison. The PSNR of SRI and MintRoute is decreased in an inverse proportion to the number of sending nodes, because the number of dropped packets increased in proportion to the number of sending nodes as shown in Figure 10 (A). MintRoute shows the lowest PSNR, because MintRoute does not care about the image quality. With MintRoute, any packets could be dropped in case of congestion, and thus, image quality decreased very much. Although SRI shows 100% of image reception in Figure 10 (B), PSNR ias decreased drastically, but shows higher PSNR than MintRoute because SRI guaranteed the packets with lower frequency by assigning higher priority. SRI could reconstruct the image with lower resolution image even if some packets were lost. Therefore, with SRI, image reception ratio was high and PSNR is higher than MintRoute. On the other hand, RAIT showed uniform PSNR because with RAIT no packet was lost during transmission.

#### 4.2.4. Energy Consumption Comparison

**Figure 13.**
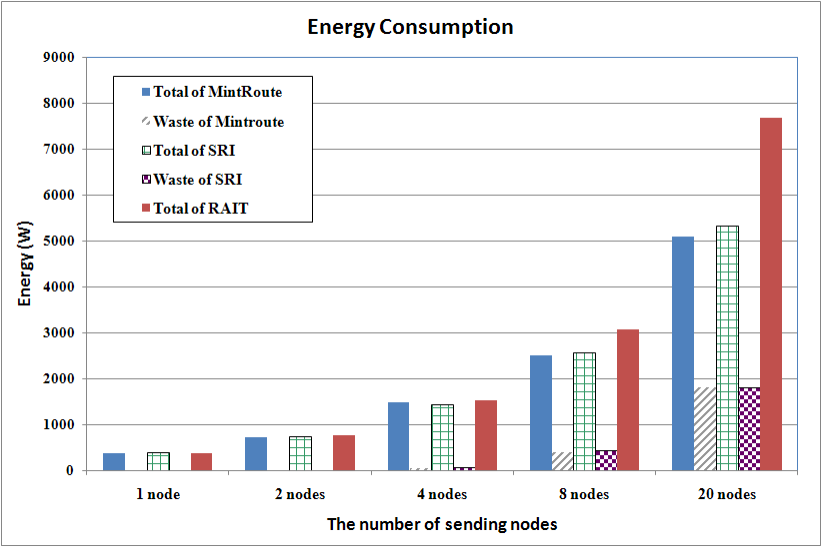
Energy Consumption. RAIT consumed more energy than SRI and MintRoute, however a large amount of energy was wasted with SRI and MintRoute.

Figure 13 presents the energy consumption comparison. With RAIT, the total amount of energy consumption was higher than that with MintRoute and SRI, because all the packets were transferred to sink nodes with RAIT, whereas some packets were lost during transmission with MintRoute and SRI. When more packets are delivered to the sink nodes, more energy is consumed and less energy is wasted. With RAIT, no packet was lost during transmission, no energy was wasted. Therefore, although the total amount of consumed energy is increased with RAIT, the energy efficiency is increased. On the other hand, with MintRoute and SRI, many packets were lost during transmission and thus the total amount of consumed energy was less than RAIT, however, wasted energy was higher than RAIT due to the packet drop. In summary, we could say that RAIT guarantees the reliable transfer of images with high energy efficiency and without energy waste.

### 4.3. Variation of Time Synchronization

#### 4.3.1. Reception Ratio

Because many applications in wireless sensor networks require the simultaneous sensing of data, much recent research has addressed the subject of time synchronization. A time synchronization protocol attempts to precisely synchronize the clocks of all sensor nodes. If sensor nodes are synchronized precisely, they will sense and send data simultaneously, which can in turn cause network congestion and packet loss. In the case of images, many packets need to be sent for a single image, so that much more congestion can take place and many more packets can be lost. In this simulation, we varied the difference in the clocks of the sensor nodes in a range from 0 s to 40 s. A clock difference of 0 s means that all sensor nodes are perfectly synchronized, and a clock difference of more than 1 s means that each sensor node begins to work and to sense randomly within the range of clock difference. 20 sensor nodes take images and send them to a sink node.

**Figure 14.**
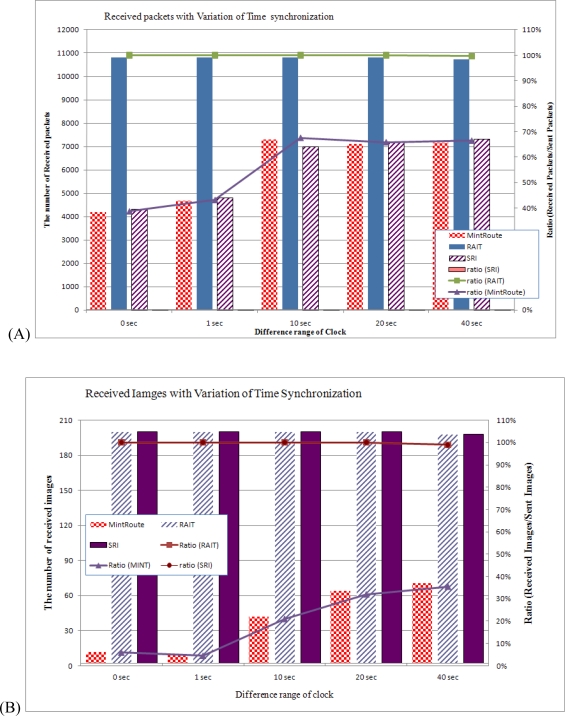
Number of received packets (A) and number of received images (B) with variations in time synchronization.

Figure 14 shows the number of received packets and images with variations of time synchronization. In Figure 14 (A), using MintRoute, only 4,200 out of 10,800 packets arrived at the sink nodes when all sensor nodes were perfectly synchronized (0 s). In this case, all of the 20 image sensor nodes simultaneously tried to send an image to a sink node, which highly increased the probability of congestion, leading to the dropping of packets in the relaying nodes. As a consequence, just 38% of the packets were received by the sink node. As the range of difference between the clocks of the sensor nodes was increased, there was an increase in the number of received packets, but when the difference between clocks exceeded 10 s, only 66% of the sent packets arrived at the sink nodes, regardless of the clock difference. In this case, even though the sensor nodes tried to send an image with a difference of 40 s in the case of little congestion among the leaf nodes, packets from several sensor nodes could cause congestion near the sink nodes. If the sensor nodes are precisely synchronized, congestion can take place around the leaf nodes of a tree while congestion can still be taking place around the sink nodes. In Figure 14 (B), with MintRoute, very few images arrived at the sink nodes when the sensor nodes were precisely synchronized. When the clock difference was 0 s, only 12 out of 200 sent images were received by a sink node, which was just 6%. In this case, we could say that most of the images were lost. Even if the clock differences were increased, the ratios of received images would be less than 40%.

With RAIT, on the other hand, all of the sent packets arrived at a sink node, for a 100% delivery rate. With this result, we would say that RAIT can prevent the congestion that took place both around the leaf nodes and around the sink nodes in order to provide completely reliable image transfer.

#### 4.3.2. Transmission duration

**Figure 15.**
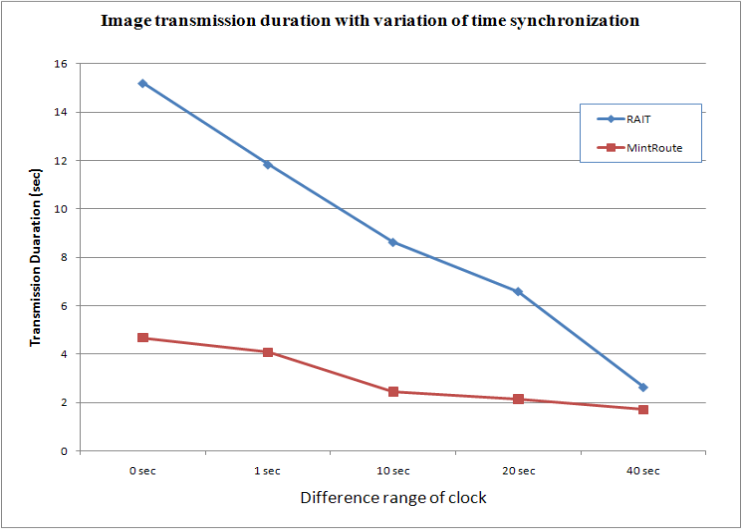
Image transmission duration with variations in time synchronization.

The precisely synchronized transmission of images from several sensor nodes can increase congestion, which can in turn lead to an increase in transmission duration. Figure 15 shows the image transmission duration with variations in the time synchronization. With RAIT, it took about 15 s for an image to be delivered from a source node to a sink node, and this duration decreased drastically as the clock differences were increased. A long delay in the case of precise synchronization might present a problem in some applications, such as emergency programs, while certain delays might be tolerable in other applications. Consequently, we could say that RAIT is suitable for applications that are not sensitive to delay but that require the reliable transfer of images.

## 5. Conclusions

Recent advances in multimedia hardware and the widespread use of wireless sensor nodes have fostered the development of wireless multimedia sensor networks. A wireless multimedia sensor network is able to ubiquitously acquire multimedia content such as images or audio from the environment.

Multimedia content whose characteristics include large size and a correlation between the data requires reliability in transmission. However, the main focus of existing solutions is on network efficiency, which are tolerant of data loss, are not appropriate for the transmission of multimedia data.

Here we have proposed a reliable asynchronous image transfer protocol, RAIT. RAIT applies a double sliding window method to node-to-node image transfer to prevent packet loss caused by network congestion. One sliding window for the receiving queue is used to prevent packet loss caused by communication failure between nodes, and another sliding window for the sending queue prevents packet loss caused by network congestion. The routing node prevents packet loss and guarantees favorable coordination between the nodes by preemptively scheduling the packets for an image. RAIT implements the double sliding window method by means of a cross-layer design between the RAIT layer, routing layer, and queue layer. Experiments have demonstrated that RAIT guarantees a higher reliability of image transmission compared to the existing protocol.
